# Diversity and Virulence of *Diaporthe* Species Associated with Peach Trunk Diseases in China

**DOI:** 10.3390/plants13223238

**Published:** 2024-11-18

**Authors:** Ying Zhou, Wei Zhang, Pranami D. Abeywickrama, Zhizheng He, Zhixiang Zhang, Yonghua Li, Shifang Li, Zaifeng Fan, Jiye Yan

**Affiliations:** 1 State Key Laboratory for Biology of Plant Diseases and Insect Pests, Institute of Plant Protection, Chinese Academy of Agricultural Sciences, Beijing 100193, China; zhouying@ippbaafs.cn (Y.Z.); zhzhxiang2003@163.com (Z.Z.); 2 Beijing Key Laboratory of Environment Friendly Management on Fruit Diseases and Pests in North China, Key Laboratory of Environment Friendly Management on Fruit and Vegetable Pests in North China (Co-Construction by Ministry and Province), Ministry of Agriculture and Rural Affairs, Institute of Plant Protection, Beijing Academy of Agriculture and Forestry Sciences, Beijing 100097, China; zhwei1125@163.com (W.Z.); pranamiabeywickrama@yahoo.com (P.D.A.); 1873039722z@gmail.com (Z.H.); lyhsierra@foxmail.com (Y.L.); 3 College of Plant Protection, China Agricultural University, Beijing 100193, China; fanzf@cau.edu.cn

**Keywords:** fungal trunk diseases, new host records, pathogenicity, *Prunus persica*, stone fruits, taxonomy

## Abstract

Peach (*Prunus persica* L.) is one of the most important and oldest stone fruits grown in China. Though *Diaporthe* species have more commonly been reported as plant pathogens, endophytes and saprophytes with a wide range of plant hosts, little is known about the *Diaporthe* species associated with peach trunk diseases in China. In the present study, forty-four *Diaporthe* isolates were obtained from trees with peach branch canker, shoot blight and gummosis symptoms in four provinces in China. Based on a combination of morphology and multi-locus sequence analysis of the rDNA internal transcribed spacer region (ITS), calmodulin (*cal*), translation elongation factor 1-α (*tef1*) and β-tubulin (*tub2*), these *Diaporthe* isolates were assigned to four species. Detailed descriptions and illustrations of all of the species, *D. arecae, D. caulivora*, *D. discoidispora* and *D*. *eres*, are provided. This study further reports the first host association of *D*. *caulivora* and *D. discoidispora* on peaches worldwide. The pathogenicity experiment results revealed that *D. arecae* was the most aggressive species, whereas *D. discoidispora* was the least aggressive on detached peach shoots. This study provides new insights into the fungi associated with peach trunk diseases in China, and the results of this study may help to facilitate routine diagnosis and planning of suitable plant disease management strategies.

## 1. Introduction

Stone fruit trees occupy an important position in the cultivation of deciduous fruit trees. They are rich in substances such as polyphenols (anthocyanins and hydroxy acids), sugars, vitamins, minerals, etc., that provide health benefits [1]. With global climate change and the abnormal occurrence of extreme weather, in recent years, sudden natural disasters such as low-temperature freezes, hailstorms, and strong winds have occurred frequently in fruit tree planting areas in China. Meanwhile, many types of major plant diseases have also occurred, and the healthy and sustainable development of the fruit tree industry has been seriously affected [2].

The peach (*Prunus persica* L.) is an important stone fruit that belongs to the family Rosaceae. It was the most valuable stone fruit in 2020 (USD 17,984,373,000 in 2020), with a combined global cultivation area of approximately 1,530,000 ha and an annual production of 24.27 million tons across the world (FAOSTAT 2022). China is the top peach-producing country, with a production of 16 million tons in 2020, which accounted for 66% of the global production (FAOSTAT 2022). However, severe yield losses can occur due to many abiotic and biotic factors. Among the various peach pests and diseases, fungal species are the dominant causative agents of diseases [3]. Fungal trunk diseases (FTDs) have been a significant threat to the global stone fruit industry. FTDs are caused by a consortium of wood-decaying fungi. These fungi colonize woody tissues, causing cankers, dieback and other decline-related symptoms in host plants [4]. Peach trunk diseases including gummosis, stem canker, twig canker, twig necrosis and shoot blight have been commonly observed worldwide, particularly in regions where stress factors are prevalent [5,6]. Zhou et al. reported the diverse fungi including Didymellaceae, Botryosphaeriaceae and Togniniaceae that are associated with peach trunk diseases in China [7].

*Diaporthe* Nitschke belongs to the family Diaporthaceae, the order Diaporthales and the class Sordariomycetes [8,9,10,11]. Their species are found worldwide on a diverse range of host plants as endophytes, pathogens and saprobes [12]. As plant pathogens, *Diaporthe* species are causing economically important diseases including root and fruit rots, dieback, cankers, leaf spots, blights, decay and wilt [13,14,15,16]. *Diaporthe* species have also been identified as pathogens that cause several diseases in many stone fruit trees [4,17]. The present study was carried out to identify and characterize the diversity of *Diaporthe* species associated with peach trunk diseases in Chinese peach orchards.

## 2. Materials and Methods

### 2.1. Sampling and Fungal Isolation

From 2020 to 2023, diseased peach samples showing symptoms of canker, shoot blight and gummosis on twigs, branches and trunks were collected from major peach-cultivation regions in four provinces of China; Beijing, Guizhou, Jilin and Sichuan.

To isolate the causative fungal pathogens, collected disease samples were treated with the following procedures. Tissue pieces taken from the margin of healthy and diseased areas were cut into small pieces (1–3 mm thick). These tissue pieces were then surface sterilized by washing them with 75% ethanol for 30 s and then rinsing them in distilled water three times. Surface-sterilized tissue pieces were then air-dried for 2–5 min in a laminar flow cabinet. Once adequately dried, tissue pieces were placed on potato dextrose agar (PDA; 200 g potato, 20 g dextrose, and 20 g agar per L) plates and incubated at 25 °C. After 5–7 days of incubation, hyphal tips growing from the margins of tissue pieces were transferred into new PDA plates and incubated for 30–40 days until they produced fruiting bodies. Pure cultures were obtained through both single hyphal tip and single spore isolation [18].

All the pure cultures obtained in this study were deposited in the culture collection of the Institute of Plant Protection, Beijing Academy of Agriculture and Forestry Sciences (JZB), Beijing, China and the culture collection numbers are given in Supplementary Table S1.

### 2.2. Morphological and Cultural Characterization

The pure cultures of fungal isolates were incubated on PDA in the dark at 25 °C to observe the colony morphology and conidial characteristics of *Diaporthe* species. The growth rate of the colony was observed after 5–7 days of incubation, and colony colours were recorded according to the colour charts of Rayner after 7 days of growth on PDA in the dark at 25 °C [19]. Digital images of morphological structures were observed and recorded by using an AxioCam 506 colour Imager Z2 photographic microscope (Carl Zeiss Microscopy, Oberkochen, Germany). Measurements of morphological features including the length and width of conidia (n = 30–40 per isolate) were taken using ZEN PRO 2012 (Carl Zeiss Microscopy).

### 2.3. DNA Extraction, PCR Amplification, and Sequencing

Fresh mycelia were scraped from the isolates grown on PDA for seven days at 25 °C and collected into 1.5 mL centrifuge tubes. Genomic DNA was extracted using a TIANcombi DNA Lyse&Det PCR Kit (TIANGEN Biotech Co., Ltd., Beijing, China) by following the manufacturer’s protocols. The gene regions of ITS, *cal*, *tef1* and *tub2* were amplified by using primer pairs ITS4/ITS5 [20,21], CAL-228F/CAL-737R [22], EF1-688F/EF1-1251R [23] and Bt2a/Bt2b [24], respectively.

Polymerase chain reaction (PCR) was performed in a BIORAD 1000^TM^ thermal cycler with a total volume of 25 μL. The PCR mixture contained 12.5 μL of 2× Taq PCR MasterMix (Taq DNA Polymerase: 0.05 units/µL; MgCl_2_: 4 mM; dNTPs: 0.4 mM; Beijing Biomed Gene Technology Co., Ltd., Beijing, China), 10.5 μL of ddH_2_O, 0.5 μL each of forward and reverse primer (10 μmol/L; Sangon Biotech, Shanghai, China) and 1 μL of DNA template (20 μg/μL). The PCR conditions were as follows: 95 °C for 3 min, followed by 34 cycles of denaturation at 95 °C for 30 s, annealing at a suitable temperature for 30 s, extension at 72 °C for 1 min and a final elongation step at 72 °C for 10 min. The annealing temperature for each gene region was 58 °C for ITS, 55 °C for *cal*, 54 °C for *tef1*, and 56 °C for *tub2*. The PCR amplification was confirmed on 1% agarose electrophoresis gels stained with ethidium bromide and visualized under UV light using Gel Doc XR + Molecular Imager Imaging system (Bio-RAD, California, USA). The amplified PCR fragments were sent to a commercial sequencing provider and sequenced by Beijing Qingke Biotechnology Co., Ltd. (Beijing, China). All sequences generated in this study were deposited in GenBank (Supplementary Table S1).

### 2.4. Phylogenetic Analyses

For all the sequences obtained in this study, chromatograms of sequence reads were checked with Bio Edit 7.0.9.0 to confirm the sequence quality. Newly generated sequences were then searched against in the NCBI (National Center for Biotechnology Information) BLASTn v. 2.15.0 (Basic Local Alignment Search Tool, https://blast.ncbi.nlm.nih.gov/Blast.cgi, accessed on 16 March 2024) for preliminary species identification. Reference sequences of the studied gene regions of *Diaporthe* strains were downloaded from GenBank (https://www.ncbi.nlm.nih.gov/genbank/, accessed on 10 April 2024) and used for phylogenetic analyses (Supplementary Table S2). The sequence alignment of each gene region was aligned with MAFFT v. 7 (https://mafft.cbrc.jp/alignment/server/, accessed on 10 May 2024). Manually trimming was carried out by using BioEdit 7.0.9.0 when necessary.

Phylogenetic analyses were conducted based on concatenated loci for the *D. arecae* species complex, *D. eres* species complex and the remaining species. The maximum likelihood (ML) analyses were accomplished using RAxML–HPC2 on XSEDE (8.2.12) [25] on the CIPRES Science Gateway platform [26] with 1000 nonparametric bootstrapping replicates, and GTR + GAMMA as the nucleotide evolution model. Bayesian inference (BI) was performed in MrBayes v.3.2.7a [27] on the XSEDE tool in the CIPRES Gateway. The evolutionary model was tested by using jModelTest2 on XSEDE in the CIPRES Gateway. MrBayes analyses were run for 1,000,0000 generations, sampling the trees at every 100th generation. From the 10,000 trees obtained, the first 2000 representing the burn-in phase were discarded. The remaining 8000 trees were used to calculate posterior probabilities in the majority rule consensus tree. The isolates obtained in this study were marked in red in the phylograms. Maximum likelihood bootstrap support values greater than 70% (BT) and Bayesian posterior probabilities (PPs) greater than 0.90 are shown at the nodes.

### 2.5. Pathogenicity Test

The pathogenicity of the selected fungal isolates (see Supplementary Table S1) obtained in this study was tested using detached green shoots of the *P. persica* cultivar Beijing No. 40 by following the methods described in Manawasinghe et al. and Chen et al. [17,28]. Healthy, 30–50 cm long, green shoots were obtained from a peach orchard in Beijing, China.

The collected peach shoots were pruned, washed with running tap water, and surface-disinfected before being used in the pathogenicity test. Surface sterilization was carried out as described in Chen et al. [17]. The selected fungal isolates grown on PDA at 25 °C for five days were used for the inoculation. A wound (5 mm diam.) was made on the surface sterilized green peach shoots using a sterilized scalpel. Agar plugs (5 mm diam.) containing mycelium from selected *Diaporthe* isolates were placed on the wound. The inoculated wounds were wrapped with Parafilm (BEMIS, USA) to prevent drying and contamination. Control shoots were inoculated with non-colonized PDA plugs. The experiment was organized with 10 replicates for each isolate. The pathogenicity test was repeated three times in the same controlled environment.

The inoculated shoots and controls were maintained at 28 °C in a growth chamber under artificial light (12/12 h light/dark cycle) at 80% relative humidity (RH). Disease symptoms were checked daily for four weeks following inoculation, and the presence of lesions advancing beyond the original 0.5 cm diameter inoculation point was considered indicative of pathogenicity. At the end of the experiment, pieces of tissue taken from the lesion area were transferred to PDA plates to re-isolate the pathogen. The significance of differences in the lesion lengths between the treatments was determined by one-way ANOVA, and the means were compared using Duncan’s multiple range test at the 5% confidence level. SPSS software v. 17 (SPSS Inc., Chicago, IL, USA) was used for the statistical analyses.

## 3. Results

### 3.1. Fungal Isolation

In the present study, a total of 44 *Diaporthe* isolates were obtained from the samples showing canker, shoot blight and gummosis symptoms collected in the four provinces of China. Twenty-three isolates from Beijing, nine isolates from Sichuan, six isolates from Jilin, and six isolates from Guizhou province were collected and most isolates were obtained from Beijing. Among them, 37 isolates were obtained from twigs, branches and trunks with canker symptoms, three fungal isolates were associated with shoot blight, one isolate with gummosis symptoms and three with trunk canker combined with gummosis. The details of *Diaporthe* isolates obtained in this study are shown in Supplementary Table S1.

### 3.2. Molecular Phylogeny

#### 3.2.1. Phylogenetic Analysis for *Diaporthe arecae* Species Complex

The combined dataset of the ITS, *cal*, *tef1* and *tub2* sequence alignments consisted of 1971 characters (456 for ITS, 423 for *cal*, 350 for *tef1* and 739 for *tub2*, including alignment gaps).

**Figure 1:**
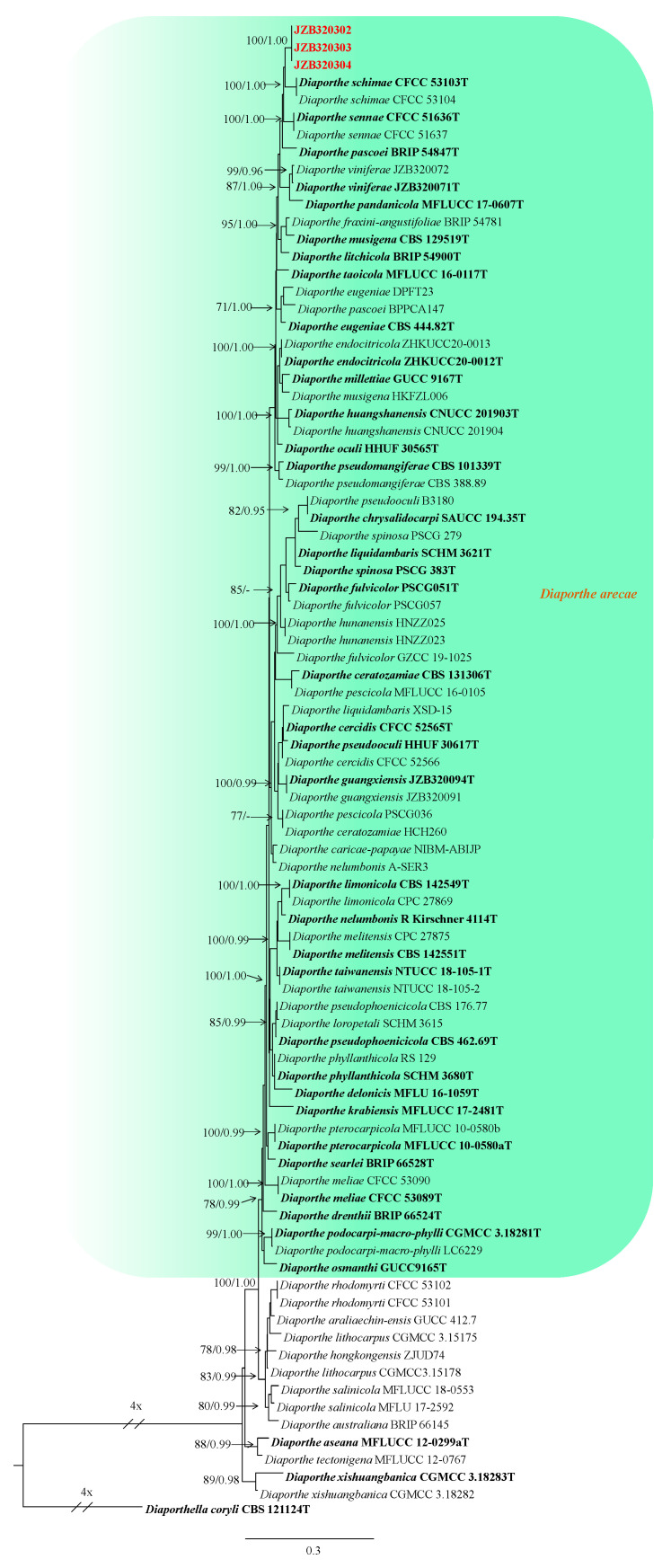
Phylogenetic tree generated from maximum likelihood analysis based on combined ITS, *cal*, *tef1* and *tub2* sequence data for the *Diaporthe arecae* species complex. Bootstrap support values for maximum likelihood (ML-BS ≥ 70%) and Bayesian posterior probabilities (PP ≥ 0.90) are shown at the nodes. Type strains are indicated in bold. The scale bar represents the expected number of changes per site. The tree is rooted with *Diaporthella coryli* (CBS 121124). Isolates obtained from this study are marked in red.

TIM2ef+I+G was determined to be the best model for ITS, TrN+G was determined to be the best model for *cal* and TPM2uf+G was determined to be the best model for *tef1*, and GTR+I+G for the *tub2* dataset. *Diaporthella coryli* (CBS 121124) was used as the outgroup taxon. The best-scoring ML tree with the final likelihood value of −13,509.648959 is shown in Figure 1. The matrix had 843 distinct alignment patterns, with 28.01% undetermined characters or gaps. The parameters for the model of the combined dataset were as follows: estimated base frequencies; A = 0.222888, C = 0.312405, G = 0.233188, T = 0.231519; substitution rates AC = 1.444031, AG = 3.239871, AT = 1.040252, CG = 0.863194, CT = 5.202729, GT = 1.000000; gamma distribution shape parameter α = 0.404970. In the phylogenetic analysis for the *Diaporthe arecae* species complex, three of the isolates obtained in this study were clustered with reference strains of *D. arecae* with good statistical support (78 ML-BS and 0.99 BI-PP) (Figure 1).

#### 3.2.2. Phylogenetic Analysis for *Diaporthe eres* Species Complex

**Figure 2:**
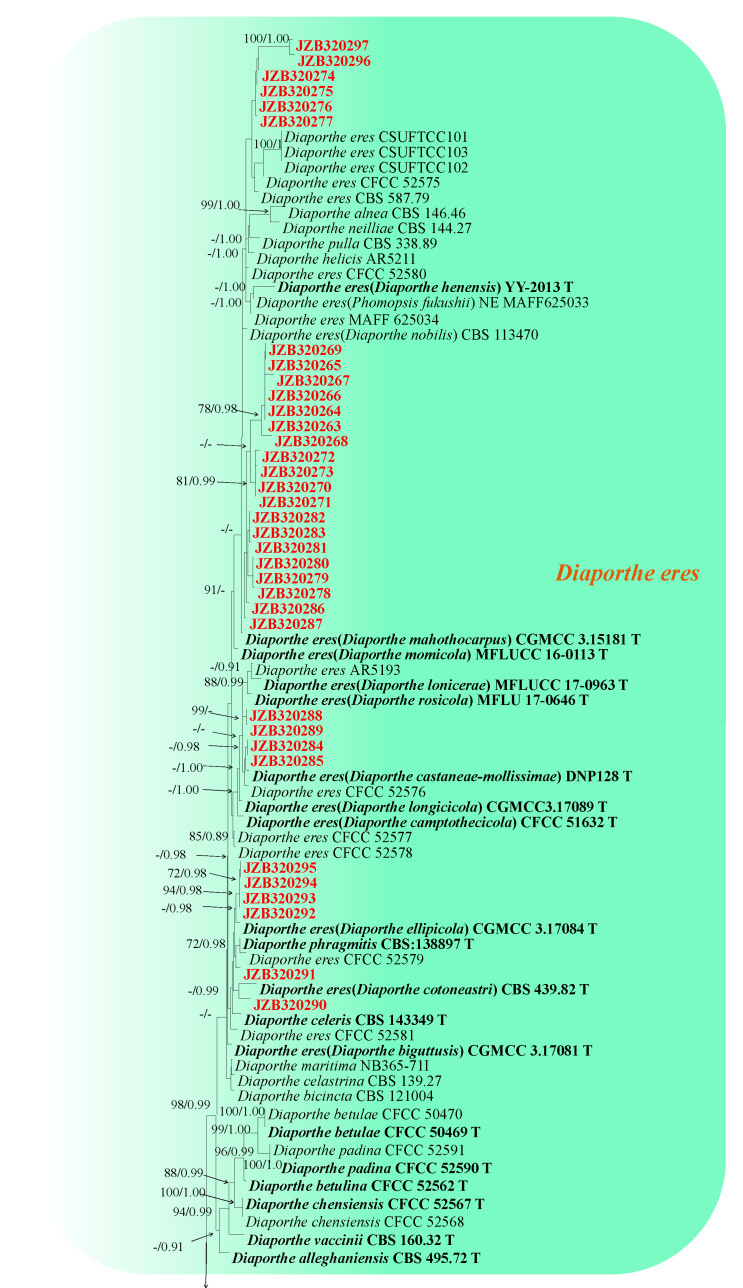

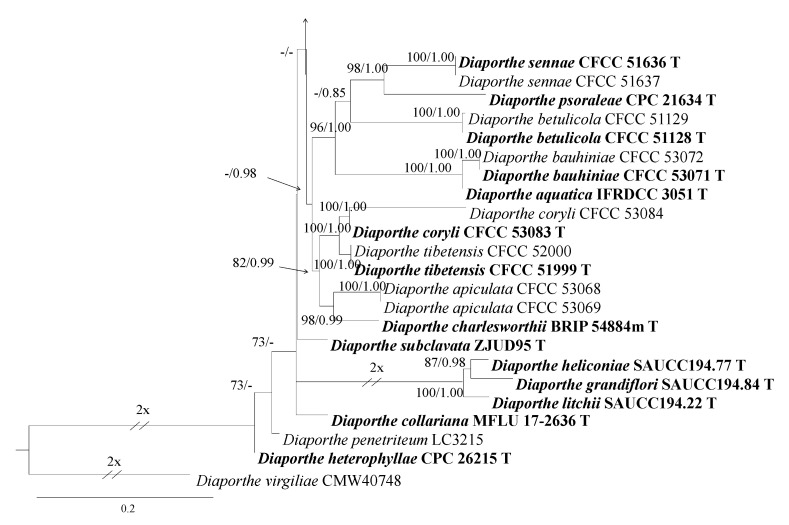
Phylogenetic tree generated from maximum likelihood analysis based on combined ITS, *cal*, *tef1* and *tub2* sequence data for the *Diaporthe eres* and related species. Bootstrap support values for maximum likelihood (ML-BS ≥ 70%) and Bayesian posterior probabilities (PP ≥ 0.90) are shown at the nodes. Type strains are indicated in bold. The scale bar represents the expected number of changes per site. The tree is rooted with *Diaporthe virgiliae* (CMW40748). Isolates obtained from this study are marked in red.

The combined dataset of the ITS, *cal*, *tef1* and *tub2* sequence alignments consisted of 1593 characters (447 for ITS, 443 for *cal*, 295 for *tef1* and 388 for *tub2*, including alignment gaps). TIM2+I+G was determined to be the best model for ITS, HKY+I+G was determined to be the best model for *cal*, TIM2+G was determined to be the best model for *tef1* and TPM3uf+G for the *tub2* dataset. *Diaporthe virgiliae* (CMW40748) was used as the outgroup taxon. The best-scoring ML tree with the final likelihood value of −14,062.066358 is given in Figure 2. The matrix had 805 distinct alignment patterns, with 14.16% of undetermined characters or gaps. Parameters for the model of the combined dataset were as follows: estimated base frequencies A = 0.215871, C = 0.323089, G = 0.234797, T = 0.226244; substitution rates AC = 1.153852, AG = 3.064092, AT = 1.104141, CG = 0.884587, CT = 3.971980, GT = 1.000000; gamma distribution shape parameter α = 0.502713.

Based on the multi-locus phylogeny, among the total isolates obtained in this study, thirty-five isolates were clustered with reference strains of *D. eres* with high statistical support (98 ML-BS and 0.99 BI-PP) (Figure 2).

#### 3.2.3. Phylogenetic Analysis for Other *Diaporthe* Species

The combined dataset of the ITS, *cal*, *tef1* and *tub2* sequence alignments consisted of 1598 characters (446 for ITS, 368 for *cal*, 415 for *tef1*, and 369 for *tub2*, including alignment gaps). TIM2+I+G was determined to be the best model for ITS, HKY+I+G was determined to be the best model for *cal*, TPM2uf+I+G was determined to be the best model for *tef1* and HKY+I+G for the *tub2* dataset. *Diaporthe eres* (CBS 587.79) was used as the outgroup taxon.

**Figure 3:**
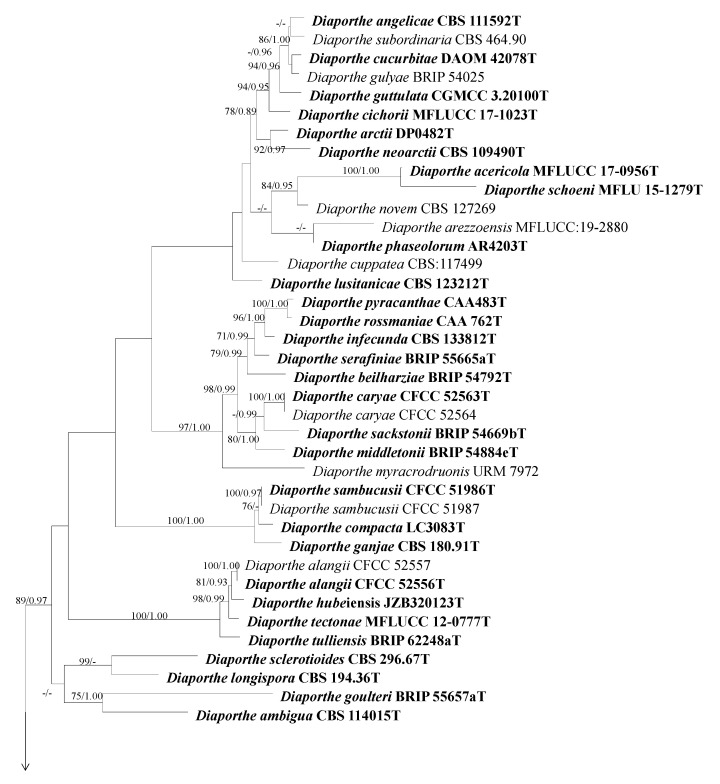

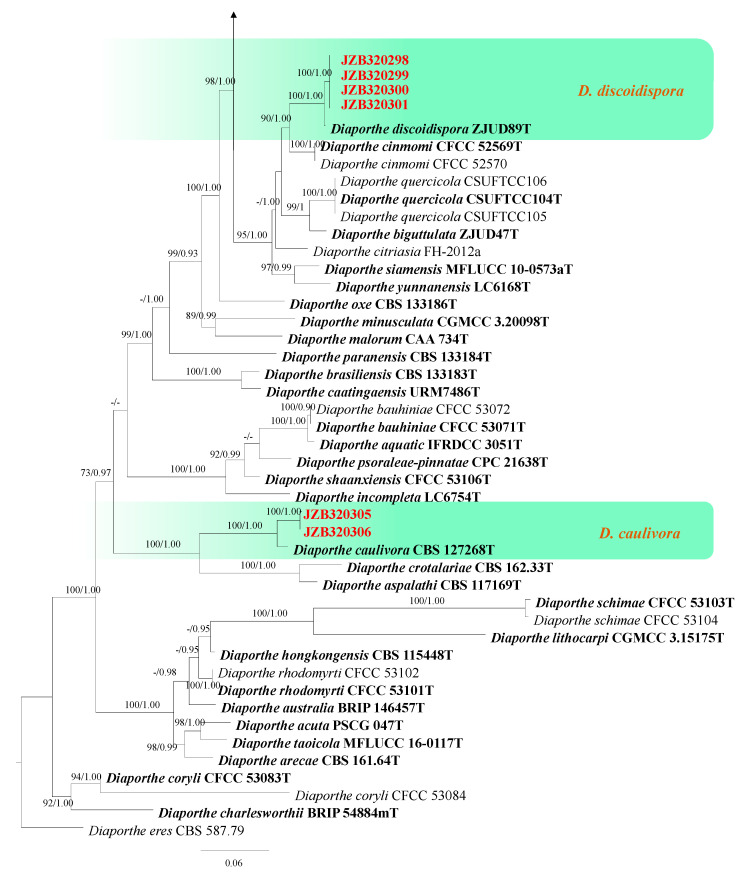
Phylogenetic tree generated from maximum likelihood analysis based on combined ITS, *cal*, *tef1* and *tub2* sequence data for the *Diaporthe* species. Bootstrap support values for maximum likelihood (ML-BS ≥ 70%) and Bayesian posterior probabilities (PP ≥ 0.90) are shown at the nodes. Type strains are indicated in bold. The scale bar represents the expected number of changes per site. The tree is rooted with *Diaporthe eres* (CBS 587.79). Isolates obtained from this study are marked in red.

The best-scoring ML tree with the final likelihood value of −21,347.849069 is given in Figure 3. The matrix had 992 distinct alignment patterns, with 17.02% of undetermined characters or gaps. Parameters for the model of the combined dataset were as follows: estimated base frequencies A = 0.217005, C = 0.313694, G = 0.240977, T = 0.228324; substitution rates AC = 1.344280, AG = 3.595697, AT = 1.267281, CG = 0.996713, CT = 5.108757, GT = 1.000000; gamma distribution shape parameter α = 0.621476.

In total, six isolates were grouped into two separate clades; among them, four isolates clustered with *Diaporthe discoidispora* (ZJUD89) with statistical support from a 100 ML-BS and a 1.00 BI-PP; and two isolates clustered with *D*. *caulivora* (CBS 127268) with ML bootstrap support value (BS) and BI posterior probabilities of 100 and 1.00, respectively (Figure 3).

### 3.3. Taxonomy

**Figure 4:**
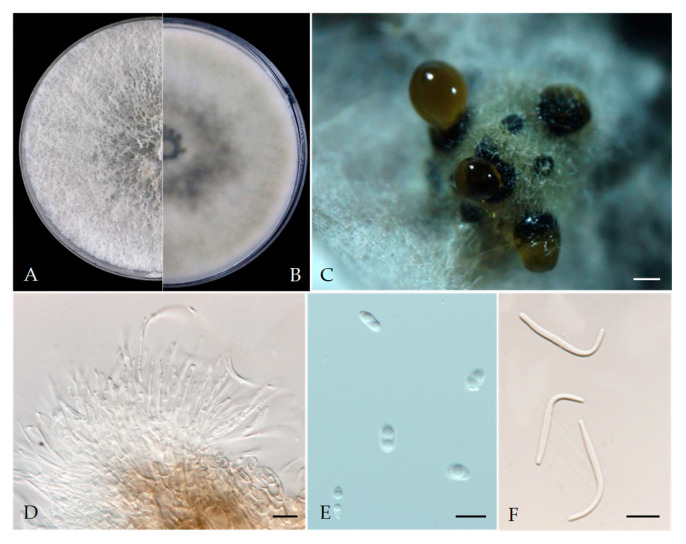
*Diaporthe arecae* (JZB320302). (**A**,**B**) Colony on PDA (front and reverse); (**C**) conidiomata on PDA; (**D**) conidiophores; (**E**) alpha conidia; (**F**) beta conidia. Scale bars: 200 μm (**C**); 10 μm (**D**–**F**).

*Diaporthe arecae* (H.C. Srivast., Zakia and Govindar.) R.R. Gomes, Glienke and Crous, (2013). Figure 4.

MycoBank: 802924.

Associated with *Prunus persica* branch canker. Sexual morph: not observed. Asexual morph: Pycnidia on PDA, dark brown to black, globose or subglobose, solitary or aggregated, embedded in the substrate, exuding a brown to black creamy mucoid conidial mass. Conidiophores 16.4–29.7 × 1.7–3.5 μm (av. = 25.2 × 2.3 μm, n = 30). Alpha conidia 6.2–9.6 × 2.5–4.6 μm (av. = 7.1 × 3.6 μm, n = 40; L/w ratio = 2.1), hyaline, aseptate, one guttulate or biguttulate, cylindrical to ellipsoid, smooth. Beta conidia 20.5–38.6 × 1.1–2.1 μm (av. = 30.2 × 1.6 μm, n = 40), filiform, hamate, tapering towards ends, hyaline, aseptate. Gamma conidia not observed.

Culture characteristics: Colonies on PDA were white and turned to grey with time, showing a white aerial mycelium; reverse: grey and dark pigmentation at the centre, covering the whole Petri plate after 5 days at 25 °C in the dark.

Material examined—China, Sichuan Province, Mianyang City, from branch canker of *Prunus persica*, September 2021, Y Zhou, living cultures JZB320302–JZB320304.

Notes: In the phylogenetic analysis of the present study, three *Diaporthe* isolates (JZB320302–JZB320304) recovered from branch canker symptoms were grouped into *Diaporthe arecae* species complex with well-supported statistical values (Figure 1). In the phylogenetic tree (Figure 1), our isolates formed a sister clade to the *D. arecae* isolates CFCC 53103 and CFCC53104 (syn. *D. schimae*). Recently, the *Diaporthe arecae* species complex was revised by Pereira et al. [29] and many previously described species have been synonymized as *D. arecae* [29,30]. Morphologically, our isolates also showed a close affinity to the *D. arecae* strains CFCC 53103 (syn. *D. schimae*) and CFCC 53104. However, we observed that our isolates developed a wider size of alpha conidia (6.2–9.6 × 2.5–4.6 μm) than CFCC 53103 (8–8.5 × 2.5–3 μm) and shorter beta conidia (20.5–38.6 × 1.1–2.1 μm) than CFCC 53103 (27.5–38.5 μm) [31].

**Figure 5:**
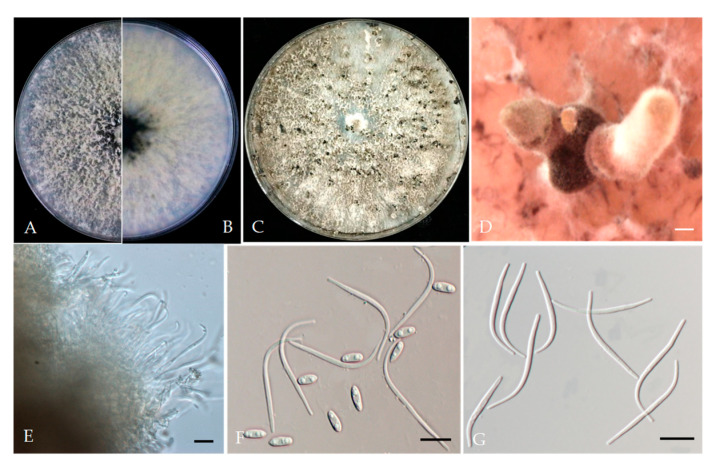
*Diaporthe eres* (JZB320287) (**A**,**B**) Colony on PDA (front and reverse) (**C**) Culture on PDA and conidiomata (**D**) conidiomata on PDA (**E**) Conidiophores (**F**) Alpha and Beta conidia (**G**) Beta conidia. Scale bars: 200 μm (**D**); 10 μm (**E**–**G**).

*Diaporthe eres* Nitschke, Pyrenomycetes Germanici, (1870). Figure 5.

MycoBank: 172057.

Associated with *Prunus persica* branch canker and gummosis. Sexual morph: not observed. Asexual morph: Conidiomata pycnidial, formed on PDA, dark brown to black, solitary or aggregated, embedded in the substrate. Conidiophores lining the inner cavity were hyaline and reduced to conidiogenous cells. Alpha conidia 6.1–9.5 × 1.8–3.4 (av. = 8.0 × 2.6 μm, n = 40; L/w ratio = 3.0), hyaline, aseptate, ovate to ellipsoidal, often biguttulate, Beta conidia 23.1–34.2 × 1.2–1.9 (av. = 28.59 × 1.5 μm, n = 40), filiform, aseptate, hyaline. Gamma conidia not observed.

Culture characteristics: Colonies on PDA were white and flat, with sparse-to-moderate aerial mycelium, then turning to pale grey; reverse: pale grey and dark pigmentation at the centre, covering the dish after 5 days at 25 °C in the dark.

Material examined: China, Beijing municipality, Haidian, Changping, Pinggu, and Shunyi districts, from Twig canker and branch canker of *Prunus persica*, July 2021, June 2022, and November 2022. Y Zhou and Z.Z He; living cultures JZB320263–JZB320277, JZB320284–JZB320287, and JZB320290–JZB320291; China, Guizhou Province, Guiyang City, Kaiyang County, from branch canker and trunk canker of *Prunus persica*, Apr. 2021. Y Zhou and Y Li, living cultures JZB320288, JZB320289, JZB320292–JZB320294, JZB320296; China, Jilin Province, Jiaohe city, from shoot blight and branch canker of *Prunus persica*, July 2022, Y Zhou, living cultures JZB320278–JZB320283. China, Sichuan Province, Mianyang City, from branch canker of *Prunus persica*, September 2021. Y Zhou and JH Jiang, living cultures JZB320295, JZB310297.

Notes: *Diaporthe eres* have been identified as a plant pathogen causing leaf spots and stem cankers in many economically important plant hosts [31,32]. *Diaporthe eres* is listed as a pathogen with plant health inspection and quarantine significance [31,33]. In the phylogenetic analysis of the present study, thirty-five isolates were grouped within the *D*. *eres* species complex. Compared with other species in the present study, *D. eres* was the most frequently isolated *Diaporthe* species from peach canker and also the only species isolated from the gummosis specimens.

**Figure 6:**
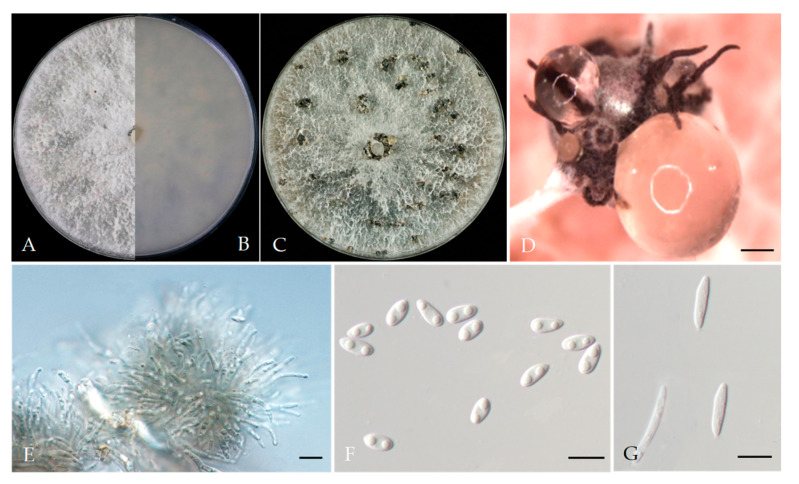
*Diaporthe caulivora* (JZB320306). (**A**,**B**) Colony on PDA (front and reverse). (**C**) Culture on PDA and conidiomata. (**D**) Conidiomata on PDA. (**E**) Conidiophores. (**F**) Alpha conidia. (**G**) Gamma conidia. Scale bars: 200 μm (**D**); 5 μm (**E**–**G**).

*Diaporthe caulivora* (Athow and Caldwell) J.M. Santos, Vrandecic and A.J.L. Phillips, (2011). Figure 6.

MycoBank: 518520.

Basionym: *Diaporthe phaseolorum* var. *caulivora*. Athow and Caldwell, (1954).

Associated with *Prunus persica* branch canker. Sexual morph: not observed. Asexual morph: Pycnidia on PDA, dark brown to black, globose, subglobose or conical, solitary or aggregated, embedded in the substrate, form long pycnidial beaks, whitish translucent conidial drops exuding from pycnidia. Conidiophores 12.4–28.4 × 1.7–3.0 μm (av. = 20.3 × 2.1 μm, n = 30). Alpha conidia 5.3–7.5 × 2.5–3.8 μm (av. = 6.5 × 3.1 μm, n = 40; L/w ratio = 2.1), hyaline, aseptate, biguttulate, spindle to ellipsoidal. Gamma conidia 9.1–20.7 × 1.6–2.9 μm (av. = 13.15 × 2.0 μm, n = 40), filiform, aseptate, straight or curve, hyaline. Beta conidia not observed.

Culture characteristics: Colonies on PDA were white and turned grey with time. White aerial mycelium; reverse: grey and dark pigmentation at the centre, covering the dish after 5 days at 25 °C in the dark.

Material examined: China, Beijing municipality, Changping Districts, from branch canker of *Prunus persica*, Aug. 2021. Y Zhou, living cultures JZB320305, JZB320306.

Notes: In this study, we obtained two isolates and based on morpho-molecular phylogenetic analysis they were identified as *Diaporthe caulivora*. Santos et al. described the sexual morph of *D*. *caulivora* and in this study, we described the asexual morph [8]. *Diaporthe caulivora* (syn. *Diaporthe phaseolorum* var. *caulivora*) was first reported as a pathogen-causing soybean stem canker in the USA in the 1980s [34]. This species has been reported to occur on *Abutilon theophrasti* from Croatia in 2005 [35]. Santos et al. showed that *D. phaseolorum* var. *caulivora* is a distinct species (*D. caulivora*) that also occurred on soybeans in Croatia in 2011 [8]. Later this species has been reported to cause postharvest fruit rot on apples in China [36]. Here, in this study, we reported the novel host association of *Diaporthe caulivora* on *Prunus persica* in China.

**Figure 7:**
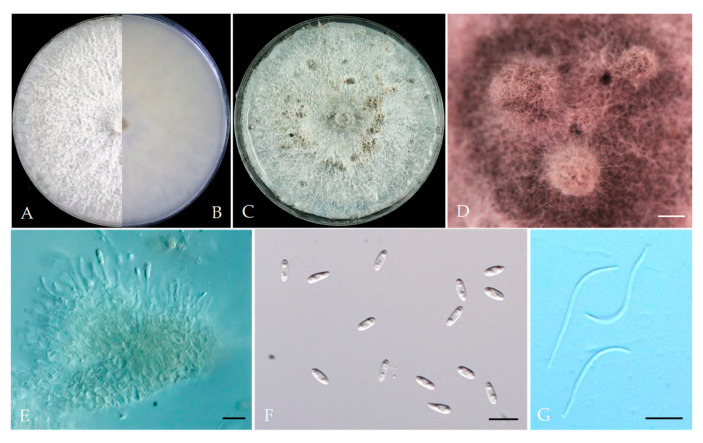
*Diaporthe discoidispora* (JZB320298) (**A**,**B**) Colony on PDA (front and reverse). (**C**) Culture on PDA and conidiomata. (**D**) Conidiomata on PDA. (**E**) Conidiophores. (**F**) Alpha conidia. (**G**) Beta conidia. Scale bars: 200 μm (**D**); 10 μm (**E**–**G**).

*Diaporthe discoidispora* F. Huang, K.D. Hyde and H.Y. Li, (2015). Figure 7.

MycoBank: 810580.

Associated with *Prunus persica* branch canker. Sexual morph: not observed. Asexual morph: Pycnidia on PDA, dark brown to black, globose, subglobose or conical, solitary or aggregated, embedded in the substrate, whitish translucent to cream conidial drops exuding from the ostioles. Conidiophores 6.5–21 × 1.8–2.8 μm (av. = 10.8 × 2.1 μm, n = 30). Alpha conidia 6.3–8.4 × 2.1–3.1 μm (av. = 7.2 × 2.7 μm, n = 40; L/w ratio = 2.7), hyaline, aseptate, biguttulate, ellipsoidal or clavate, base subtruncate. Beta conidia 25.6–38 × 1.1–2.0 μm (av. = 32.1 × 1.5 μm, n = 40), abundant in culture, filiform, straight or hamate, hyaline, eguttulate, aseptate. Gamma conidia not observed.

Culture characteristics: Colonies on PDA were white and turned to grey with time, white aerial mycelium; reverse: grey and dark pigmentation at the centre, covering the dish after 5 days at 25 °C in the dark.

Material examined: China, Sichuan Province, Mianyang City, from branch canker of *Prunus persica*, September 2021. Y Zhou, living cultures JZB320298—JZB320301.

Notes: In the phylogenetic analysis of the present study, four isolates recovered from branch canker of peach from Sichuan province in China and they were clustered together with *Diaporthe discoidispora* (Figure 1). Further, these isolates (JZB320298, JZB320299, JZB320300, and JZB320301) were morphologically similar to the type species of *D. discoidispora* (ZJUD89). They are similar in terms of their conidial size, whereas alpha conidia’s size is 6.3–8.4 × 2.1–3.1 μm (JZB320298), and 5.6–8 × 2.1–3.2 μm (ZJUD89), beta conidia’s size is 25.6–38 × 1.1–2.0 μm (JZB320298), and 21.2–38.7 × 0.9–1.6 μm (ZJUD89). *Diaporthe discoidispora* was first isolated from a non-symptom twig of *Citrus unshiu* in China [37]. Later, this species was reported to be associated with *Camellia* from China [38]. Here, in this study, we first reported the novel host association of *Diaporthe discoidispora* on *Prunus persica* in China.

### 3.4. Pathogenicity Test Results

**Figure 8:**
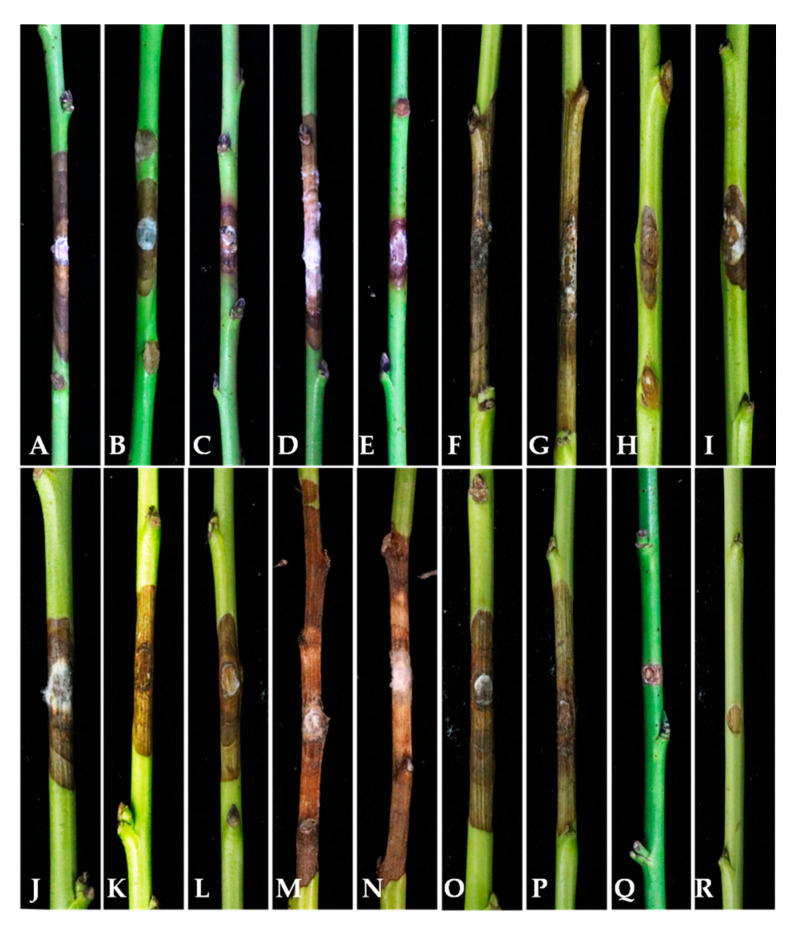
Disease lesions developed on peach green shoots after 7 days of inoculation of *Diaporthe* isolates. (**A**–**J**) *D. eres*; (**A**): JZB320279; (**B**): JZB320280; (**C**): JZB320281; (**D**): JZB320282; (**E**): JZB320283; (**F**): JZB320287; (**G**): JZB320288; (**H**): JZB320290; (**I**): JZB320293; (**J**): JZB320295. (**K**,**L**) *D. discoidispora*; (**K**): JZB320298; (**L**): JZB320299. (**M**,**N**) *D. arecae*; (**M**): JZB320302; (**N**): JZB320303. (**O**,**P**) *D. caulivora*; (**O**): JZB320305; (**P**): JZB320306. (**Q**,**R**): control.

**Figure 9:**
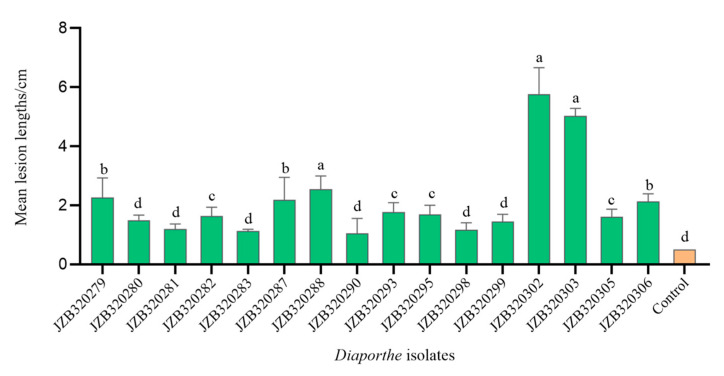
Average lesion length (cm) resulting from inoculation trials with *Prunus persica* L. on the seventh day. Vertical bars represent the standard error of means. Different letters above the bars indicate treatments that were significantly different (*p* = 0.05). *Diaporthe eres*: JZB320279, JZB320280, JZB320281, JZB320282, JZB320283, JZB320287, JZB320288, JZB320290, JZB320293, JZB320295; *D*. *discoidispora*: JZB320298, JZB320299, *D. arecae*: JZB320302, JZB320303. *D*. *caulivora*: JZB320305, JZB320306.

In this study, a total of 16 isolates (see Supplementary Table S1) belonging to four *Diaporthe* species were tested for their pathogenicity on detached *Prunus persica* shoots. Ten isolates of *D. eres* (JZB320279–JZB320283, JZB320287, JZB320288, JZB320290, JZB320293, JZB320295), and six isolates from all other *Diaporthe* species identified in this study, were used for this experiment. Three days after inoculation, all *Diaporthe* isolates were showing symptoms on detached peach shoots, including necrotic lesions similar to the disease symptoms observed in the field. Larger necrotic lesions along the bark were observed after seven days of inoculation. The length of the lesion was measured on the seventh day after inoculation. The symptom development (Figure 8) and the average lesion length (Figure 9) are shown below.

Based on the results of the pathogenicity test, all the *Diaporthe* species identified in this study showed pathogenicity towards the detached green shoots of *Prunus persica*. Among them, *Diaporthe arecae* showed the highest lesion development, followed by *D. caulivora*, and *D. eres*. *Diaporthe discoidispora* showed the lowest lesion development on the peach green shoots.

## 4. Discussion

The present study reveals the diversity and pathogenicity of *Diaporthe* species associated with *Prunus persica* trunk diseases in China. In total, 44 isolates were obtained and they were classified into four *Diaporthe* species using morphological and multi-loci phylogenetic data. In this study, we identified four previously described *Diaporthe* species named, *D. arecae*, *D. caulivora*, *D. discoidispora* and *D. eres* from *Prunus persica* in China.

Among the *Diaporthe* species obtained, *D. eres* was the most frequently isolated, representing 79% of the isolates recovered from diseased branch samples. *Diaporthe eres* was described by Nitschke on *Ulmus* sp. collected in Germany. *Diaporthe eres* has been reported to be a weak to moderate pathogen of woody plants [32,37,38,39,40,41,42,43]. Several studies have demonstrated that this species is a weak pathogen or an opportunistic saprobe of grapevines in various geographic regions [40,44,45]. Other than on grapevines, *D. eres* has been reported on *Aralia elata*, *Camellia*, citrus, peach and pear, causing dieback in China [37,38,39,46,47]. Based on the results of the pathogenicity experiment, we observed variations in the aggressiveness of our isolates of *D. eres* on detached peach shoots. The pathogenicity of these isolates needs to be analyzed further with field experiments to check their ability to cross-infect other stone fruit crops in the field. According to the results of isolation frequency and the pathogenicity test, we rank *D. eres* as the dominant pathogen of the genus *Diaporthe* on peach trunk diseases in China, which is consistent with previous research [47,48].

*Diaporthe arecae* (syn. *Subramanella arecae*) was first introduced in 1962 and associated with severe post-harvest fruit rot of the areca nut (*Areca catechu* L.) in India [49]. *Diaporthe arecae* species complex was first designated by Huang et al. [37], who isolated 13 endophytic strains from *Citrus* spp. in different provinces of China that were clustered in a poorly supported clade with the ex iso type strain of *D. arecae*. In recent studies, Pereira et al. presented updated molecular analyses for the *D. arecae* complex based on the Genealogical Concordance Phylogenetic Species Recognition (GCPSR) principle and Poisson Tree Processes (PTPs) coalescent models. These analyses provided strong evidence that all species previously described in the *D. arecae* subclade are conspecific [29]. Dissanayake et al. divided species in the *Diaporthe* genus into several specific sections based on phylogenetic analyses that can avoid the construction of lengthy phylogenetic trees of the entire genus in future taxonomic studies, *D. arecae* was restructured into the section Foeniculina [30].

In China, *Diaporthe taoicola* isolate MFLUCC 16-0117 was previously reported to be associated with the dieback of peaches in 2017 [47]. According to recent analyses by Pereira et al. and Dissanayake et al. [29,30], *D. taoicola* (MFLUCC 16-0117) has been synonymized into *D. arecae*. In this study, *D. arecae* isolates obtained from the samples collected from Sichuan, showed the strongest pathogenicity; thus, attention should be given to surveying the development trends of diseases in local orchards.

*Diaporthe discoidispora* was first isolated from a healthy twig of *Citrus unshiu* from China in 2015 [37] and was subsequently found to be weakly aggressive towards the tested citrus varieties. In this study, we extended its host range to peach and confirmed it has a pathogenicity towards the peach. Two tested *D. discoidispora* isolates in this study were equally aggressive on peach shoots, similar to most isolates of *D. eres*.

*Diaporthe caulivora,* previously known as *D. phaseolorum var. caulivora*, was an important pathogen of soybean across the world. Santos et al. proposed that *D*. *phaseolorum var. caulivora* should be raised to species status and the name *D*. *caulivora* was introduced to accommodate it in 2011 [8]. We tested the pathogenicity of *D*. *caulivora,* and found that it was more aggressive than *D. discoidispora* and half of the isolates of the *D. eres*.

The discovery of these species of *Diaporthe* from *Prunus persica* in China and their worldwide occurrence indicates the polyphagous and cosmopolitan nature of the species within this genus. Conducting complementary studies based on multi-loci sequencing of *Diaporthe* species is crucial to back up reliable species identification. The descriptions and molecular data of *Diaporthe* species presented in this study will act as a resource for plant pathologists, plant quarantine officials and taxonomists for better identification of *Diaporthe* and its species boundaries. Such studies are requisite to explore this group of fungi in different unexploited biomes, to disclose the degree of diversity and to uphold more appropriate control measures to prevent their spread. The present study recorded two *Diaporthe* species, *D. discoidispora* and *D. caulivora* associated with peach branch disease for the first time. The results presented herein offer opportunities for several fields, including peach breeding for disease-resistant cultivars, screening for new fungicides and devising appropriate quarantine and management strategies to prevent and control diseases.

## 5. Conclusions

The trunk diseases of peach are a poorly documented disease that negatively affects peach production and cultivation in China. The diverse fungal species are known to be associated with this disease worldwide, and this present study represents the investigation of *Diaporthe* species associated with trunk diseases of peach in China. Based on the combination of morphology and multi-locus sequence analysis, a total of 44 *Diaporthe* isolates were assigned to four species named, *D. arecae, D. caulivora*, *D. discoidispora* and *D. eres*. All the *Diaporthe* species showed pathogenicity towards the peach shoots and *D. arecae* was the most aggressive species. Given the global economic significance of peaches, more investigations related to the role of phytopathogens are needed. Even though no cure is known for these stone fruit fungal trunk diseases, integrated disease management **practices** are recommended. The results presented in this study provide inputs for several disciplines, including quarantine and disease management strategies for peach trunk diseases.

## Data Availability

All the sequence data generated in this study were available in the NCBI GenBank and the following accession numbers are given in Supplementary Table S1.

## Supplementary Materials

The following supporting information can be downloaded at: https://www.mdpi.com/article/10.3390/plants13223238/s1, Table S1: Details of the *Diaporthe* isolates obtained in this study; Table S2. GenBank accession numbers of the sequences used for phylogenetic analysis in this study.
