# Supplementary material for: Diversity and Virulence of Diaporthe Species Associated with Peach Trunk Diseases in China
Source: Plants (Basel). 2024 Nov 18;13(22):3238. doi: 10.3390/plants13223238 (PMC11597932; doi:10.3390/plants13223238)
Supplement: Supplementary file 1 [file plants-13-03238-s001.zip › plants-3278244-supplementary.pdf]

Table S1. Details of the *Diaporthe* isolates obtained in this study

Species	Culture Collection number / Isolates	Location	Sample symptom	GenBank accession numbers			
				ITS	*cal*	*tef1*	*tub2*
Diaporthe arecae	JZB320302*	Chengdu, Sichuan	Branch canker	PQ210990	PQ222189	PQ222198	PQ222206
D. arecae	JZB320303*	Chengdu, Sichuan	Branch canker	PQ210991	PQ222190	PQ222199	PQ222207
D. arecae	JZB320304	Chengdu, Sichuan	Branch canker	PQ210992	PQ222191	NA	PQ222208
D. caulivora	JZB320305*	Changping, Beijing	Twig canker	PQ210993	PQ222192	PQ222200	PQ222209
D. caulivora	JZB320306*	Changping, Beijing	Twig canker	PQ210994	PQ222193	PQ222201	PQ222210
D. discoidispora	JZB320298*	Chengdu, Sichuan	Branch canker	PQ210986	PQ222185	PQ222194	PQ222202
D. discoidispora	JZB320299*	Chengdu, Sichuan	Branch canker	PQ210987	PQ222186	PQ222195	PQ222203
D. discoidispora	JZB320300	Chengdu, Sichuan	Branch canker	PQ210988	PQ222187	PQ222196	PQ222204
D. discoidispora	JZB320301	Chengdu, Sichuan	Branch canker	PQ210989	PQ222188	PQ222197	PQ222205
D. eres	JZB320263	Pinggu, Beijing	Twig canker	NA	NA	NA	PQ093749
D. eres	JZB320264	Pinggu, Beijing	Twig canker	PQ060381	NA	PQ093717	PQ093750
D. eres	JZB320265	Pinggu, Beijing	Twig canker	PQ060382	NA	PQ093718	NA
D. eres	JZB320266	Pinggu, Beijing	Twig canker	NA	PQ093687	PQ093719	PQ093751
D. eres	JZB320267	Pinggu, Beijing	Twig canker	PQ060383	PQ093688	PQ093720	PQ093752
D. eres	JZB320268	Pinggu, Beijing	Twig canker	PQ060384	PQ093689	PQ093721	PQ093753
D. eres	JZB320269	Pinggu, Beijing	Twig canker	PQ060385	PQ093690	PQ093722	PQ093754
D. eres	JZB320270	Pinggu, Beijing	Twig canker	PQ060386	PQ093691	PQ093723	PQ093755
D. eres	JZB320271	Pinggu, Beijing	Twig canker	PQ060387	PQ093692	PQ093724	PQ093756
D. eres	JZB320272	Pinggu, Beijing	Twig canker	PQ060388	NA	PQ093725	PQ093757
D. eres	JZB320273	Pinggu, Beijing	Twig canker	PQ060389	PQ093693	PQ093726	PQ093758
D. eres	JZB320274	Pinggu, Beijing	Trunk canker	PQ060390	PQ093694	PQ093727	PQ093759
D. eres	JZB320275	Pinggu, Beijing	Trunk canker	PQ060391	PQ093695	PQ093728	PQ093760
D. eres	JZB320276	Pinggu, Beijing	Trunk canker	PQ060392	PQ093696	PQ093729	PQ093761
D. eres	JZB320277	Pinggu, Beijing	Trunk canker	PQ060393	PQ093697	NA	PQ093762
D. eres	JZB320278	Jiaohe, Jilin	Shoot blight	PQ060394	PQ093698	PQ093730	PQ093763
D. eres	JZB320279*	Jiaohe, Jilin	Shoot blight	PQ060395	PQ093699	PQ093731	PQ093764
D. eres	JZB320280*	Jiaohe, Jilin	Shoot blight	PQ060396	PQ093700	PQ093732	PQ093765
D. eres	JZB320281*	Jiaohe, Jilin	Trunk gummosis	PQ060397	PQ093701	PQ093733	PQ093766
D. eres	JZB320282*	Jiaohe, Jilin	Trunk gummosis	NA	PQ093702	PQ093734	PQ093767
D. eres	JZB320283*	Jiaohe, Jilin	Trunk gummosis	PQ060398	PQ093703	PQ093735	PQ093768
D. eres	JZB320284	Shunyi, Beijing	Trunk canker	NA	PQ093704	PQ093736	NA
D. eres	JZB320285	Shunyi, Beijing	Trunk canker	NA	PQ093705	PQ093737	PQ093769
D. eres	JZB320286	Shunyi, Beijing	Trunk canker	NA	PQ093706	PQ093738	PQ093770
D. eres	JZB320287*	Changping, Beijing	Twig canker	PQ060399	PQ093707	NA	PQ093771
D. eres	JZB320288*	Guiyang, Guizhou	Trunk canker	PQ060400	PQ093708	PQ093739	PQ093772
D. eres	JZB320289	Guiyang, Guizhou	Trunk canker	PQ060401	NA	PQ093740	PQ093773
D. eres	JZB320290*	Haidian, Beijing	Twig canker	PQ060402	PQ093709	PQ093741	PQ093774
D. eres	JZB320291	Changping, Beijing	Branch canker	PQ060403	PQ093710	PQ093742	PQ093775
D. eres	JZB320292	Guiyang, Guizhou	Branch canker	PQ060404	PQ093711	PQ093743	PQ093776
D. eres	JZB320293*	Guiyang, Guizhou	Branch canker	PQ060405	PQ093712	PQ093744	PQ093777

Species	Culture Collection number / Isolates	Location	Sample symptom	GenBank accession numbers			
				ITS	*cal*	*tef1*	*tub2*
D. eres	JZB320294	Guiyang, Guizhou	Branch canker	PQ060406	PQ093713	PQ093745	PQ093778
D. eres	JZB320295*	Chengdu, Sichuan	Branch canker	PQ060407	PQ093714	PQ093746	PQ093779
D. eres	JZB320296	Guiyang, Guizhou	Branch canker	PQ060408	PQ093715	PQ093747	PQ093780
D. eres	JZB320297	Chengdu, Sichuan	Branch canker	PQ060409	PQ093716	PQ093748	PQ093781

JZB: Culture collection at Institute of Plant Protection, Beijing Academy of Agriculture and Forestry Sciences , Beijing, China;

ITS: internal transcribed spacer regions 1 & 2 including 5.8S nrDNA gene; *cal*: calmodulin; *tef1*: Partial translation elongation factor 1-α; *tub2*: beta-tubulin;

*: the isolates used for the pathogenicity test.

N/A: denotes no sequence available.

Table S2. GenBank accession numbers of the sequences used for phylogenetic analysis in this study

Species	Isolate	GenBank accession numbers			
		ITS	*cal*	*tef1*	*tub2*
Diaporthe acericola	MFLUCC 17-0956T	KY964224	KY964137	KY964180	KY964074
D. acuta	PSCG 047T	MK626957	MK691125	MK654802	MK691225
D. alangii	CFCC 52556T	MH121491	MH121415	MH121533	MH121573
D. alangii	CFCC 52557	MH121492	MH121416	MH121534	MH121574
D. alleghaniensis	CBS 495.72T	KC343007	KC343249	KC343733	KC343975
D. alnea	CBS 146.46	KC343008	KC343250	KC343734	KC343976
D. ambigua	CBS 114015T	KC343010	KC343252	KC343736	KC343978
D. angelicae	CBS 111592T	KC343027	KC343269	KC343753	KC343995
D. anhuiensis	CNUCC 201901	MN219718	MN224549	MN224668	MN227008
D. anhuiensis	CNUCC 201902	MN219727	MN224550	MN224669	MN227009
D. apiculata	CFCC 53068	MK432651	MK442973	MK578127	MK578054
D. apiculata	CFCC 53069	MK432652	MK442974	MK578128	MK578055
D. aquatica	IFRDCC 3051T	JQ797437	NA	NA	NA
D. arctii	DP0482T	KJ590736	KJ612133	KJ590776	KJ610891
D. arecae	CBS 161.64T	KC343032	KC343274	KC343758	KC344000
D. arengae	CBS 114979T	KC343034	KC343276	KC343760	KC344002
D. arezzoensis	MFLUCC:19-2880	MT185503	NA	NA	NA
D. aspalathi	CBS 117169T	KC343036	KC343278	KC343762	KC344004
D. australiana	BRIP 66145	MN708222	NA	MN696522	MN696530
D. bauhiniae	CFCC 53071T	MK432648	MK442970	MK578124	MK578051
D. bauhiniae	CFCC 53072	MK432649	MK442971	MK578125	MK578052
D. beilharziae	BRIP 54792T	JX862529	NA	JX862535	KF170921
D. betulae	CFCC 50470	KT732951	KT732998	KT733017	KT733021
D. betulae	CFCC 50469T	KT732950	KT732997	KT733016	KT733020
D. betulicola	CFCC 51128T	KX024653	KX024659	KX024655	KX024657
D. betulicola	CFCC 51129	KX024654	KX024660	KX024656	KX024658
D. betulina	CFCC 52562T	MH121497	MH121421	MH121539	MH121579
D. bicincta	CBS 121004	KC343134	KC343376	KC343860	KC344102
D. biguttulata	ZJUD47T	KJ490582	NA	KJ490461	KJ490403
D. brasiliensis	CBS 133183T	KC343042	KC343284	KC343768	KC344010
D. caatingaensis	URM7486T	KY085927	KY115597	KY115603	KY115600
D. caryae	CFCC 52563T	MH121498	MH121422	MH121540	MH121580
D. caryae	CFCC 52564	MH121499	MH121423	MH121541	MH121581
D. caulivora	CBS 127268T	MH864501	KC343287	KC343771	KC344013
D. celastrina	CBS 139.27	KC343047	KC343289	KC343773	KC344015
D. celeris	CBS 143349T	MG281017	MG281712	MG281538	MG281190
D. cercidis	CFCC 52565T	MH121500	MH121424	MH121542	MH121582
D. cercidis	CFCC 52566	MH121501	MH121425	MH121543	MH121583
D. charlesworthii	BRIP 54884mT	KJ197288	NA	KJ197250	KJ197268

Species	Isolate	GenBank accession numbers			
		ITS	*cal*	*tef1*	*tub2*
D. chensiensis	CFCC 52568	MH121503	MH121427	MH121545	MH121585
D. chensiensis	CFCC 52567T	MH121502	MH121426	MH121544	MH121584
D. cichorii	MFLUCC 17-1023T	KY964220	KY964133	KY964176	KY964104
D. cinnamomi	CFCC 52569T	MH121504	NA	MH121546	MH121586
D. cinnamomi	CFCC 52570	MH121505	NA	MH121547	MH121587
D. citriasiana	FH-2012a	JQ954645	KC357491	JQ954663	KC357459
D. collariana	MFLU 17-2636T	MG806115	MG783042	MG783040	MG783041
D. compacta	LC3083T	KP267854	NA	KP267928	KP293434
D. coryli	CFCC 53083T	MK432661	MK442981	MK578135	MK578061
D. coryli	CFCC 53084	MK432662	MK442982	MK578136	MK578062
D. crotalariae	CBS:162.33T	MH855395	JX197439	GQ250307	KC344024
D. cucurbitae	DAOM 42078T	KM453210	NA	KM453211	KP118848
D. cuppatea	CBS:117499	MH863021	KC343299	KC343783	KC344025
D. discoidispora	ZJUD89T	KJ490624	NA	KJ490503	KJ490445
D. drenthii	CBS 146453T	MN708229	NA	MN696526	MN696537
D. eres	AR5193	KJ210529	KJ434999	KJ210550	KJ420799
D. eres	CBS 113470	KC343146	KC343388	KC343872	KC344114
D. eres	CBS 439.82	FJ889450	JX197429	GQ250341	JX275437
D. eres	CBS 587.79	KC343153	KC343395	KC343879	KC344121
D. eres	CFCC 51632	KY203726	KY228877	KY228887	KY228893
D. eres	CFCC 52575	MH121510	NA	MH121552	MH121592
D. eres	CFCC 52576	MH121511	MH121432	MH121553	MH121593
D. eres	CFCC 52577	MH121512	MH121433	MH121554	MH121594
D. eres	CFCC 52578	MH121513	MH121434	MH121555	MH121595
D. eres	CFCC 52579	MH121514	NA	MH121556	NA
D. eres	CFCC 52580	MH121515	NA	MH121557	MH121596
D. eres	CFCC 52581	MH121516	NA	MH121558	MH121597
D. eres	CGMCC 3.15181T	KC153096	NA	KC153087	KF576312
D. eres	CGMCC 3.17081	KF576282	NA	KF576257	KF576306
D. eres	CGMCC 3.17084	KF576270	NA	KF576245	KF576294
D. eres	CGMCC 3.17089	KF576267	NA	KF576242	KF576291
D. eres	CGMCC 3.17639	KC898258	NA	NA	KF600608
D. eres	CSUFTCC101	ON076564	NA	ON081656	NA
D. eres	CSUFTCC102	ON076565	NA	ON081657	NA
D. eres	CSUFTCC103	ON076566	NA	ON081658	NA
D. eres	DNP128	JF957786	KJ435040	KJ210561	KJ420801
D. eres	MAFF 625034	JQ807469	KJ435023	JQ807418	KJ420819
D. eres	MAFF625033	JQ807468	KJ435017	JQ807417	KJ420814
D. eres	MFLU 17-0646T	MG828895	NA	MG829270	MG843877
D. eres	MFLUCC 16-0113T	KU557563	KU557611	KU557631	KU557587
D. eres	MFLUCC 17-0963T	KY964190	KY964116	KY964146	KY964073
D. fraxini-angustifoliae	BRIP 54781T	JX862528	NA	JX862534	KF170920

Species	Isolate	GenBank accession numbers			
		ITS	*cal*	*tef1*	*tub2*
D. fulvicolor	PSCG 051T	MK626859	MK691132	MK654806	MK691236
D. ganjae	CBS 180.91T	KC343112	KC343354	KC343838	KC344080
D. goulteri	BRIP 55657aT	KJ197290	NA	KJ197252	KJ197270
D. grandiflori	SAUCC194.84T	MT822612	MT855691	MT855809	MT855924
D. gulyae	BRIP 54025	JF431299	NA	JN645803	KJ197271
D. guttulata	CGMCC 3.20100T	MT385950	MW022470	MT424685	MT424705
D. helicis	AR5211	KJ210538	KJ435043	KJ210559	KJ420828
D. heliconiae	SAUCC194.77T	MT822605	MT855684	MT855802	MT855917
D. heterophyllae	CPC 26215T	MG600222	MG600218	MG600224	MG600226
D. hongkongensis	CBS 115448T	KC343119	KC343361	KC343845	KC344087
D. hubeiensis	JZB320123T	MK335809	MK500235	MK523570	MK500148
D. incompleta	LC6754T	KX986794	KX999289	KX999186	KX999226
D. infecunda	CBS 133812T	KC343126	KC343368	KC343852	KC344094
D. krabiensis	MFLUCC 17-2481T	MN047100	NA	MN433215	MN431495
D. litchicola	BRIP 54900T	JX862533	NA	JX862539	KF170925
D. litchii	SAUCC194.22T	MT822550	MT855635	MT855747	MT855863
D. lithocarpus	CGMCC3.15175T	KC153104	KF576236	KC153095	KF576311
D. lithocarpus	CGMCC3.15178	KC153103	NA	KC153094	N/A
D. longispora	CBS 194.36T	MH855769	KC343377	KC343861	KC344103
D. lusitanicae	CBS 123212T	MH863279	KC343378	KC343862	KC344104
D. malorum	CAA 734T	KY435638	KY435658	KY435627	KY435668
D. maritima	NB365-71I	KU552025	NA	KU552023	KU574615
D. meliae	CFCC 53089T	MK432657	NA	ON081654	MK578057
D. meliae	CFCC 53090	MK432658	NA	ON081655	MK578058
D. middletonii	BRIP 54884eT	KJ197286	NA	KJ197248	KJ197266
D. minusculata	CGMCC 3.20098T	MT385957	MW022475	MT424692	MT424712
D. musigena	CBS 129519T	KC343143	KC343385	KC343869	KC344111
D. myracrodruonis	URM 7972	MK205289	MK205290	MK213408	MK205291
D. neilliae	CBS 144.27	KC343144	KC343386	KC343870	KC344112
D. neoarctii	CBS 109490T	KC343145	KC343387	KC343871	KC344113
D. novem	CBS 127269	KC343155	KC343397	KC343881	KC344123
D. oxe	CBS 133186T	KC343164	KC343406	KC343890	KC344132
D. padina	CFCC 52590T	MH121525	MH121443	MH121567	MH121604
D. padina	CFCC 52591	MH121526	MH121444	MH121568	MH121605
D. pandanicola	MFLUCC 17-0607T	MG646974	NA	NA	MG646930
D. pandanicola	SAUCC194.82	MT822610	MT855689	MT855922	MT855807
D. paranensis	CBS 133184T	KC343171	KC343413	KC343897	KC344139
D. pascoei	BRIP 54847T	JX862532	NA	JX862538	KF170924
D. penetriteum	LC3215	KP267879	NA	KP267953	NA
D. perseae	CBS 151.73	KC343173	KC343415	KC343899	KC344141
D. pescicola	MFLUCC 16-0105T	KU557555	KU557603	KU557623	KU557579
D. phaseolorum	AR4203T	KJ590738	KJ612135	KJ590739	KJ610893

Species	Isolate	GenBank accession numbers			
		ITS	*cal*	*tef1*	*tub2*
D. phragmitis	CBS:138897T	KP004445	NA	NA	KP004507
D. podocarpi-macrophylli	CGMCC 3.18281 T	NR_152467	NA	NA	NA
D. podocarpi-macrophylli	LC6155T	KX986774	KX999278	KX999167	KX999207
D. podocarpi-macrophylli	LC62229	KX986771	KX999277	KX999164	KX999204
D. pseudomangiferae	CBS 101339T	KC343181	KC343423	KC343907	KC344149
D. pseudophoenicicola	CBS 176.77	KC343183	KC343425	KC343909	KC344151
D. psoraleae	CPC 21634T	KF777158	NA	KF777245	KF777251
D. psoraleae-pinnatae	CPC 21638T	KF777159	NA	NA	KF777252
D. pterocarpicola	MFLUCC 10-0580aT	JQ619887	JX197433	JX275403	JX275441
D. pulla	CBS 338.89	KC343152	KC343394	KC343878	KC344120
D. pyracanthae	CAA483T	KY435635	KY435656	KY435625	KY435666
D. quercicola	CSUFTCC104T	ON076567	ON081670	ON081659	NA
D. quercicola	CSUFTCC105	ON076568	ON081671	ON081660	NA
D. quercicola	CSUFTCC106	ON076569	ON081672	ON081661	NA
D. rhodomyrti	CFCC 53101T	MK432643	MK442965	MK578119	MK578046
D. rhodomyrti	CFCC 53102	MK432644	MK442966	MK578120	MK578047
D. rossmaniae	CAA 762T	MK792290	MK883822	MK828063	MK837914
D. sackstonii	BRIP 54669bT	KJ197287	NA	KJ197249	KJ197267
D. salinicola	MFLU 17-2592	MN047099	NA	MN077074	N/A
D. salinicola	MFLUCC 18-0553	MN047098	NA	MN077073	N/A
D. sambucusii	CFCC 51986T	KY852495	KY852499	KY852507	KY852511
D. sambucusii	CFCC 51987	KY852496	KY852500	KY852508	KY852512
D. schimae	CFCC 53103T	MK432640	MK442962	MK578116	MK578043
D. schimae	CFCC 53104	MK432641	MK442963	MK578117	MK578044
D. schoeni	MFLU 15-1279T	KY964226	KY964139	KY964182	KY964109
D. sclerotioides	CBS 296.67T	MH858974	KC343435	KC343919	KC344161
D. searlei	CBS 146456T	MN708231	NA	NA	MN696540
D. sennae	CFCC 51636T	KY203724	KY228875	KY228885	KY228891
D. sennae	CFCC 51637	KY203725	KY228876	KY228886	KY228892
D. serafiniae	BRIP 55665aT	KJ197274	NA	KJ197236	KJ197254
D. shaanxiensis	CFCC 53106T	MK432654	MK442976	MK578130	NA
D. siamensis	MFLUCC 10-0573aT	JQ619879	JX197423	JX275393	JX275429
D. spinosa	PSCG 383T	MK626849	MK691129	MK654811	MK691234
D. subclavata	ZJUD95T	KJ490630	NA	KJ490509	KJ490451
D. subordinaria	CBS 464.90	KC343214	KC343456	KC343940	KC344182
D. taoicola	MFLUCC 16-0117T	KU557567	NA	KU557635	KU557591
D. tectonae	MFLUCC 12-0777T	KU712430	KU749345	KU749359	KU743977
D. tibetensis	CFCC 51999T	MF279843	MF279888	MF279858	MF279873
D. tibetensis	CFCC 52000	MF279844	MF279889	MF279859	MF279874
D. tulliensis	BRIP 62248aT	KR936130	NA	KR936133	KR936132
D. vaccinii	CBS 160.32T	KC343228	KC343470	KC343954	KC344196
D. viniferae	JZB320072	MK341551	MK500119	MK500107	MK500112

Species	Isolate	GenBank accession numbers			
		ITS	*cal*	*tef1*	*tub2*
D. virgiliae	CMW 40748	KP247566	NA	NA	KP247575
D. yunnanensis	LC 6168T	KX986796	KX999290	KX999188	KX999228
Diaporthella coryli	CBS 121124T	KC343004	KC343246	KC343730	KC343972

CBS: Culture collection of the Centraalbureau voor Schimmelcultures, Fungal Biodiversity Centre, Utrecht, The Netherlands; CPC: Personal collection of P.W. Crous, Utrecht, The Netherlands;

JZB: Culture collection at Institute of Plant Protection, Beijing Academy of Agriculture and Forestry Sciences culture collection, Beijing, China;

MFLUCC: Mae Fah Luang University Culture Collection, Thailand;

ITS: internal transcribed spacer regions 1 & 2 including 5.8S nrDNA gene; *cal*: calmodulin; *tef1*: Partial translation elongation factor 1-α; *tub2*: beta-tubulin;

T: ex-type or ex-epitype culture. N/A: denotes no sequence available.
